# Imaging of the ulcerated carotid atherosclerotic plaque: a review of the literature

**DOI:** 10.1007/s13244-017-0543-8

**Published:** 2017-02-03

**Authors:** Vasileios Rafailidis, Ioannis Chryssogonidis, Thomas Tegos, Konstantinos Kouskouras, Afroditi Charitanti-Kouridou

**Affiliations:** 1 Department of Radiology, AHEPA University General Hospital, Aristotle University of Thessaloniki, Thessaloniki, Greece; 2 1st Neurological Department, AHEPA University General Hospital, Aristotle University of Thessaloniki, Thessaloniki, Greece

**Keywords:** Carotid atherosclerosis, Carotid ulcer, Ultrasound imaging, CT angiography, MRI angiography

## Abstract

**Abstract:**

Carotid atherosclerotic disease constitutes a major modern health problem whose diagnosis primarily relies on imaging. Grading of stenosis has been long used as the main factor for risk stratification and guiding of management. Nevertheless, increasing evidence has shown that additional plaque characteristics such as plaque composition and surface morphology play an important role in the occurrence of symptoms, justifying the term “vulnerable plaque”. Carotid plaque surface characteristics either in the form of surface irregularities or ulceration represent an important factor of vulnerability and are associated with the occurrence of neurologic symptoms. The delineation of the carotid plaque surface can be performed with virtually all imaging modalities including ultrasound, contrast-enhanced ultrasound, multi-detector computed tomography angiography, magnetic resonance angiography and the traditional reference method of angiography. These techniques have shown varying levels of diagnostic accuracy for the identification of ulcerated carotid plaques or plaque surface irregularities. As a consequence and given its high clinical significance, radiologists should be familiar with the various aspects of this entity, including its definition, classification, imaging findings on different imaging modalities and associations. The purpose of this review is to present the current literature regarding carotid plaque ulcerations and present illustrative images of ulcerated carotid plaques.

***Teaching Points*:**

*• Plaque surface and ulceration represent risk factors for stroke in carotid disease.*

*• Characterisation of the plaque surface and ulcerations can be performed with every modality.*

*• US is the first-line modality for carotid disease and identification of ulcerations.*

*• The administration of microbubbles increases US accuracy for diagnosis of carotid ulceration.*

*• MDCTA and MRA are valuable for diagnosing ulceration and evaluating plaque composition.*

## Introduction

Carotid atherosclerotic disease represents a well-established cause of ischaemic stroke, accounting for up to 20% of strokes or transient ischaemic attacks (TIA) [1]. Stroke constitutes a major cause of acquired disability in adults and the second most frequent cause of mortality in developed nations [2]. The degree of luminal stenosis has long been serving as the primary criterion for risk stratification of patients and treatment decision-making, being a well-known risk factor for the development of neurologic symptoms in patients with carotid disease [3]. Nevertheless, current research has concluded that plaque features other than degree of stenosis contribute to the occurrence of neurologic symptoms, justifying the introduction of the term “vulnerable plaque” [4], responsible for almost half of stroke cases [5]. From a pathogenic point of view, this is explained by the mechanism of arterio-arterial embolism describing the creation and detachment of embolic material from a plaque and its subsequent transportation to the intracranial circulation, causing vascular occlusion and occurrence of symptoms [4].

The plaque surface morphology is among those features related to the risk for embolic stroke and characterising vulnerability. Based on this criterion, carotid plaques are typically classified into smooth, irregular or ulcerated [6, 7]. The presence of ulceration itself is a well-known feature of vulnerability with high clinical significance as entailing increased risk for neurologic symptoms. As a consequence its accurate diagnosis is essential and primarily relies on imaging. Ultrasound (US) undoubtedly represents the first-line modality for both screening and initial diagnostic evaluation of carotid disease [8]. Beyond grading of stenosis with widely accepted velocity criteria [9], US is valuable in evaluating the plaque’s echogenicity and surface characteristics [10]. Digital subtractive angiography (DSA) has been deemed to be the gold standard for the evaluation of carotid disease but is interventional and has an associated risk for stroke [11]. On the other hand, the emergence and widespread availability of non-invasive cross-sectional imaging modalities such as multidetector computed tomography angiography (MDCTA) or magnetic resonance angiography (MRA) offered a valuable alternative to DSA, providing excellent spatial resolution and great accuracy for evaluation of plaque fine surface characteristics [12]. Nevertheless, the role of US has been significantly boosted by the introduction of US contrast media and recent evidence concludes that contrast-enhanced ultrasound (CEUS) contributes significantly to the characterisation of carotid plaques, in terms of both surface delineation (ulcer detection and characterisation) and internal structure (visualisation of intraplaque neovascularisation) [13, 14].

The purpose of this review is to deliver an overview of the literature regarding carotid plaque ulceration. Subjects that will be discussed include the ulcer’s definition and classification, clinical significance and imaging. Imaging findings of ulcerated carotid plaques will be illustrated with diagrammatic representations and educational images, correlating US, CEUS, MDCTA and MRA.

## Definitions

**Fig. 1:**
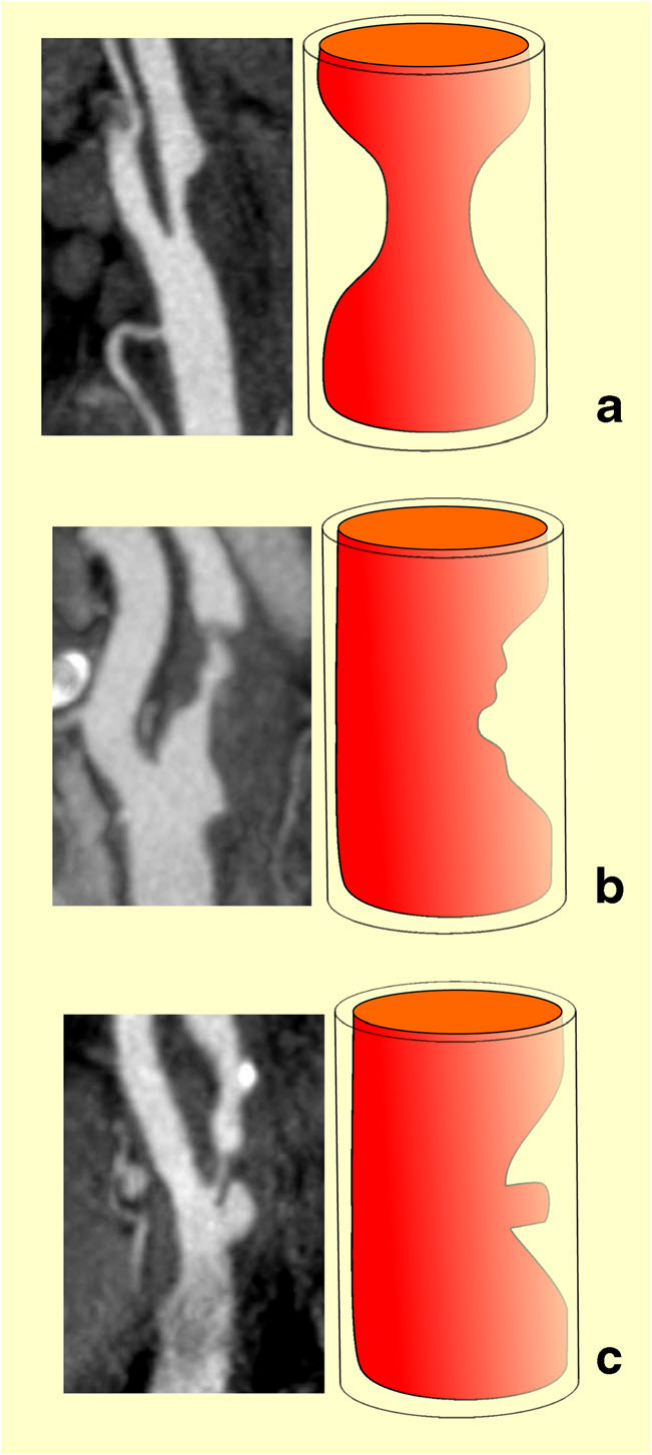
Diagrammatic representations and MDCTA images illustrating the classification of carotid plaques based on their surface morphology as smooth (**a**), irregular (**b**) and ulcerated (**c**)

The definition of carotid plaque ulceration varies depending on the modality used or even among different research groups [10, 15]. In terms of histology, the term “ulceration” describes an endothelial defect of at least 1000 μm in width, resulting in the exposure of the plaque’s necrotic core to circulation [7, 16]. From the point of view of imaging, different criteria have been used to define ulceration [10, 15]. In general, a plaque’s surface can be characterised as smooth, irregular or ulcerated [4], with smooth referring to a plaque with regular luminal morphology (Fig. 1). The term irregular can be used for plaques whose surface fluctuates from 0.3 mm to 0.9 mm [4], whereas the term ulceration is reserved for cavities measuring at least 1 mm [7, 13] or 2 mm according to different studies and proposed risk stratification systems [17, 18]. Each ulcer is characterised by a neck and a base, both of varying sizes and resulting in various shapes. De Bray et al. introduced the most widely used US criteria, stipulating that ulcerations should (1) be at least 2 mm long and deep, (2) have a well-demarcated posterior wall at its base on B-mode and (3) show internal flow reversal on colour Doppler technique [15]. According to the newer criteria, ulcerations can be diagnosed when there is evidence of a cavity on the plaque surface, irrespective of size, whose surface echogenicity is lower compared to the adjacent intimal plaque’s border on B-mode [10]. On MDCTA, an ulceration can be diagnosed when contrast medium is identified extending beyond the vascular lumen (and within the plaque limits) for at least 1 mm in at least two planes [13]. On CEUS, which is virtually an angiographic technique, the ulcer definition requires the interruption of the plaque-lumen border for at least 1 × 1 mm [13]. When it comes to 3D US, the volume criterion of a cavity measuring at least 1 mm^3^ has been used [19].

## Frequency/location of ulceration

Carotid MDCTA studies have shown that 55–62% of plaques are smooth, 15–22% irregular and 16–44% ulcerated [13, 20, 21]. The frequency of carotid ulcerations depends on the presence of symptoms and the modality used. Histological ulcerations have been reported to occur in up to 89% of cases [22]. In a study enrolling more than 3000 patients examined with DSA, the frequency of ulceration was 14% [23]. In MDCTA studies, ulceration was discovered in 10–31% of carotid plaques [7, 21, 24]. There is a documented difference of ulcer frequency between asymptomatic and symptomatic plaques with the former being ulcerated in 14% of cases and the latter in 36% [25]. Other studies have detected ulcers in 48% of symptomatic plaques and 31% of asymptomatic [26].

Studies have shown that ulcerations are more likely to affect the part of a plaque lying proximally to the point of maximum stenosis (up to 70% of cases) rather than distally. This observation is explained by the higher shear stress applied to the part of the plaque proximal to the point of maximum stenosis [7, 20, 23]. The ulcer frequency is also associated with the degree of stenosis. Namely, ulcer incidence was 16.6% for plaques causing 50–69% stenosis, 22.6% for 70–84% stenosis and 33.4% for stenosis >85% [7]. Other studies have identified a significant percentage of ulcerations in plaques causing <50% stenosis [24].

## Clinical significance of ulceration

The association of carotid ulceration and TIA was initially described in a small published group of patients in 1968 where surgical removal of the ulcerated plaque relieved ipsilateral symptoms [27, 28]. It thus became evident that a superficial ulceration may act as a risk factor for neurologic symptoms, on the pathogenic basis of arterio-arterial embolism of thrombotic material [2, 25, 27, 29]. This mechanism was highlighted by a study showing that ulcerated carotid plaques are more common in patients with TIA and that in up to 12.6% of embolic TIAs the emboli originate from such plaques [30]. The arterio-arterial embolism theory was also illustrated in the experimental setting, involving recordings of dye flow inside replicas of ulcerated carotid plaques. The dye could be seen moving in a swirling pattern within the ulcer cavity, explaining how platelet aggregates could be formed and pulled back to circulation and thus the intracranial circulation [31]. This swirling flow pattern observed within the ulcer cavity [31] explains the vortex of colours or “yin-yang” image occasionally observed within ulcers with the colour Doppler technique [32]. This pattern was also observed with CEUS where microbubbles were seen swirling inside the ulcer cavity [33]. This haemodynamic phenomenon in combination with the more frequently observed thrombosis on ulcerated plaques [25] favours the arterio-arterial embolism.

The clinical significance of ulcerated carotid plaques has been documented through numerous studies. For instance, ulceration has been correlated with embolic signals on transcranial Doppler US, regardless of its depth [34, 35], while being recognised as a risk factor for stroke [36, 37]. Namely, the risk for stroke in a patient with ulcerated carotid plaque tends to increase with the degree of stenosis, while it is 1.24 to 3.43 times greater compared to non-ulcerated plaques [37]. However, other studies have correlated ulceration with symptoms even in plaques causing low-grade stenosis (≤50%) [38]. Ulceration is more frequently found in symptomatic carotid plaques [39] and is also associated with the occurrence of new symptoms in asymptomatic patients [40]. Indeed, multiparametric analysis has confirmed the correlation of ipsilateral TIA or stroke with the presence of carotid ulceration in asymptomatic prospectively observed patients [41]. Histologically detected ulceration was found up to 2.32 times more frequently in symptomatic patients [42]. US studies have shown that ulcerated plaques are related to a seven-fold increase in ipsilateral stroke risk [43] while hypoechoic ulcerated plaques are associated with a nine-fold increase [44]. A higher risk for stroke in patients with ulcerated plaque compared to those without ulceration was also reported in the NASCET study [45]. It was recently concluded that ulceration increases the risk for neurologic symptoms by approximately four times [17]. Similarly to US, MDCTA-detected ulceration is also correlated with symptoms [7].

Even simple irregularity of the plaque surface, without a clear ulceration, has been correlated with an increased risk for stroke [36]. Such plaques have been associated with embolic vascular territory infarcts on brain CT, while smooth plaques were correlated either with normal scans or lacunar infarcts [29]. The plaque surface morphology, specifically the plaque irregularity as assessed with MDCTA, has been identified as an important risk factor for symptoms in patients with 30–69% stenosis [46]. Irregular carotid plaques as evaluated on high-resolution B-mode US have been correlated with higher risk for stroke compared with smooth plaques [47]. Nevertheless, a recent meta-analysis could not confirm these findings showing that US-detected plaque irregularity was not correlated with symptoms [17]. Finally, it was found that irregular stenosis is associated with increased risk for stroke in the long term after an index symptom [48].

## Classification of ulceration

**Fig. 2:**
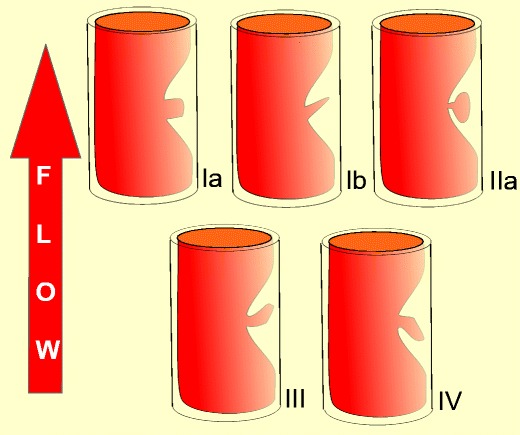
Diagrammatic representations illustrating the classification of ulcerated carotid plaques in relation to the blood flow direction

**Fig. 3:**
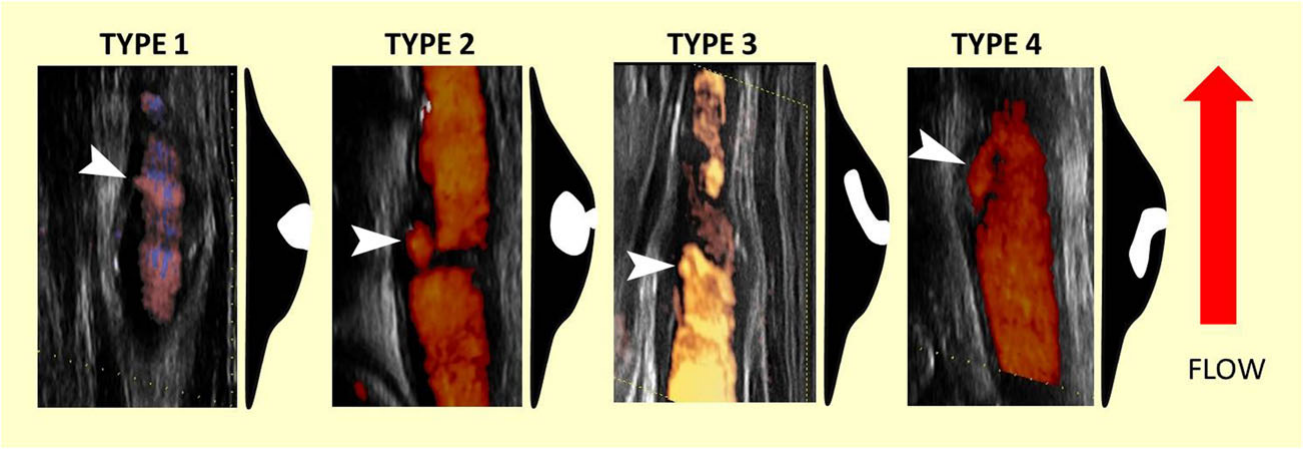
Ultrasonographic images and diagrammatic representations illustrating the various ulcer types (arrowheads showing the ulcers) (from left to right: eFlow, Power Doppler, xFLow and Power Doppler technique). eFlow and xFlow are high-definition blood flow imaging modes available in certain ultrasound devices

**Fig. 4:**
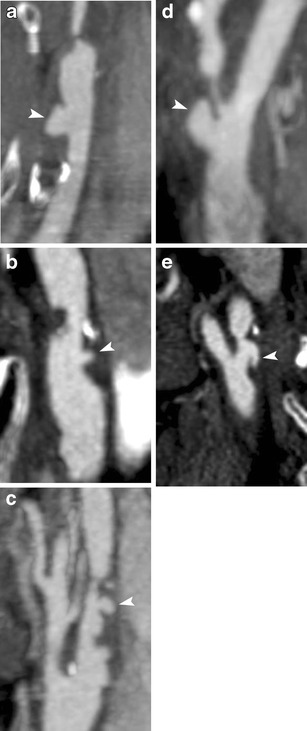
MDCTA images showing carotid plaques with ulcers of type 1a (**a**), 1b (**b**), 2 (**c**), 3 (**d**) and 4 (**e**) (arrowheads showing ulcers)

In an early study carotid plaque ulcers were classified as presented in Table 1 [49] (Figs. 2, 3 and 4).

**Table 1:** Classification of carotid ulcerations based on their morphology

Classification of carotid ulcerations based on morphology	
Type 1	Ulcer projecting perpendicular to the vessel’s lumen with parallel sides (1a) or sides converging to a point (1b)
Type 2	Narrow-necked ulcer (“mushroom shaped”) or an ulcer with no neck visible
Type 3	Ulcer with a proximal neck and its main part pointing distally, parallel to blood flow direction
Type 4	Ulcer with distal neck and its main part pointing proximally, opposite to blood flow direction

Alternatively, carotid ulcerations can be classified based on their location on the plaque surface and in relation to the point of maximum stenosis into three types, as presented in Table 2 [23].

**Table 2:** Classification of carotid ulcerations based on their location on the plaque surface

Classification of carotid ulcerations based on their location on the plaque surface
Proximal to the point of maximum stenosis
Distal to the point of maximum stenosis
Situated at the point of maximum stenosis

**Fig. 5:**
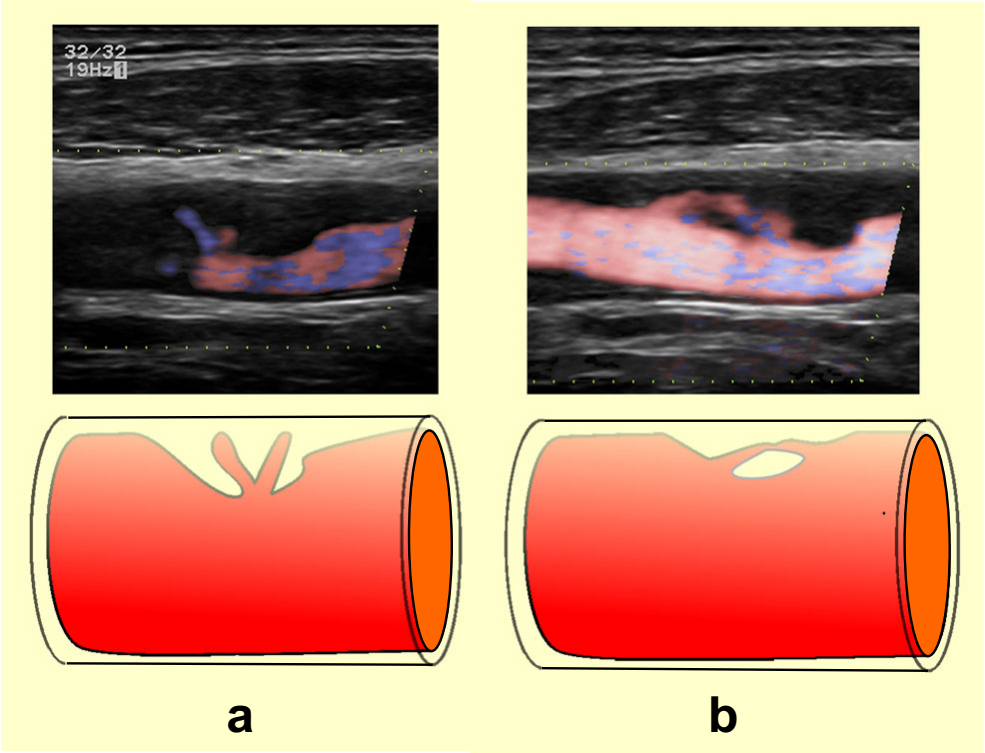
Ultrasonographic images and diagrammatic representations of potential previously undescribed types of ulcerated plaques. eFlow technique delineating a V-shaped ulcer (**a**) and an ulcer resembling a bucket handle (**b**), both in common carotid artery plaques

Nevertheless, with the introduction of modern sensitive flow visualisation techniques, new types of ulcers can be identified including V-shaped ulcers or ulcers resembling a bucket handle. Moreover, multiple ulcers can be found within the same plaque (Fig. 5).

## Composition of ulcerated carotid plaques

**Fig. 6:**
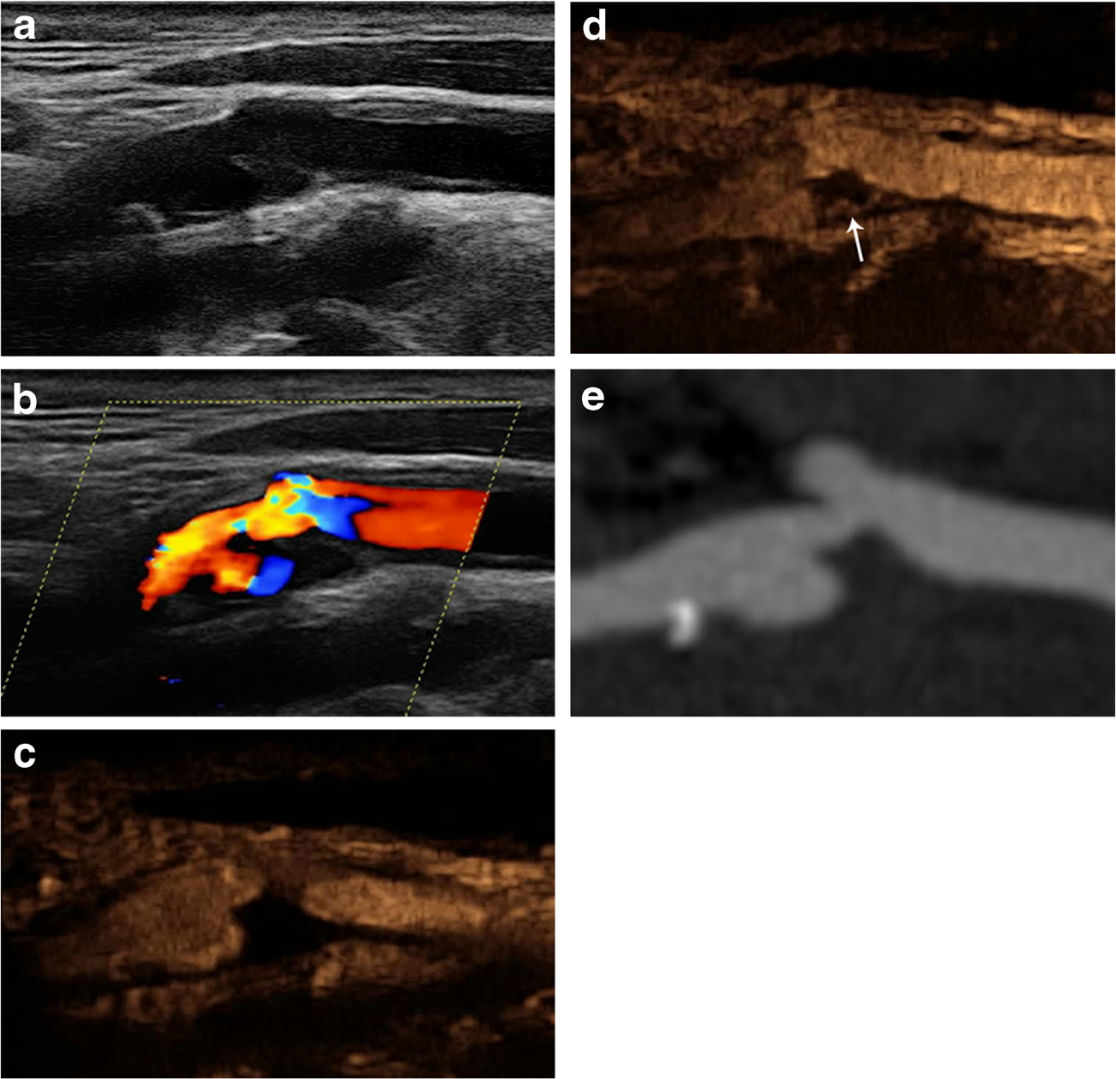
Imaging findings of an ulcerated carotid plaque with intraplaque neovascularisation. B-mode image (**a**) identified the presence of an anechoic cavity possibly representing an ulceration. Colour Doppler image (**b**) showing flow reversal within the ulcer cavity. CEUS (**c**) a few seconds after the intravenous administration of microbubbles confirms the presence of ulceration while the plaque appears anechoic. Delayed CEUS image (**d**) revealed the presence of moving microbubbles within the plaque and near its adventitial side representing intraplaque neovascularisation (*arrow*). MDCTA image (**e**) showing the ulcerated plaque in correlation with ultrasonographic techniques

**Fig. 7:**
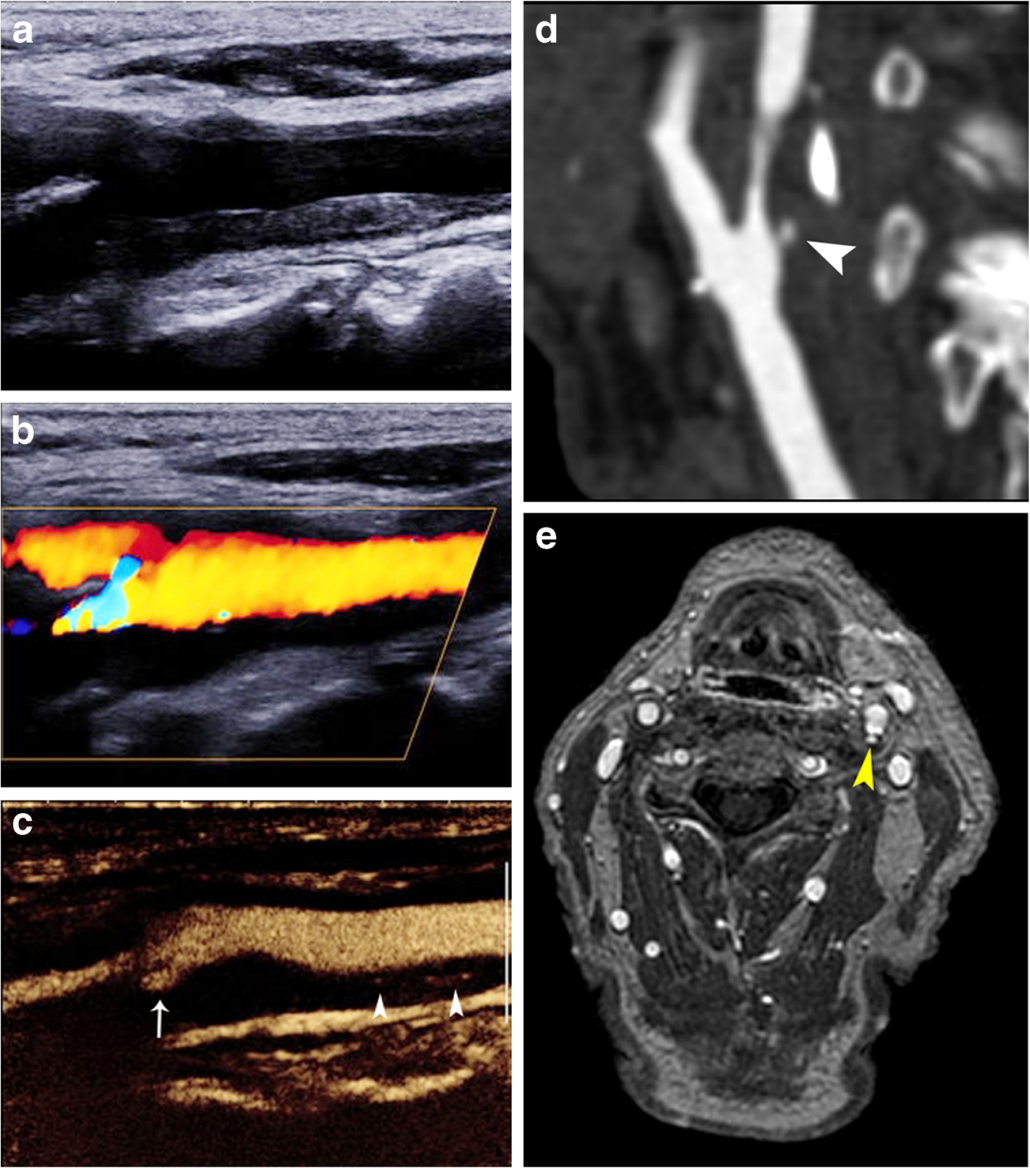
Imaging findings in a symptomatic patient with ulcerated carotid plaque. B-mode image (**a**) showing a smooth atherosclerotic plaque, appearing predominantly hypoechoic. Colour Doppler image (**b**) confirming the plaque’s smooth surface and showing severe luminal stenosis of the internal carotid artery. Note that the flow is not adequately visualised in the internal carotid artery as parts of the lumen contain no flow signals. CEUS (**c**) improved visualisation of blood flow in the whole field of view and provided detailed delineation of the plaque surface. Note the presence of a small superficial ulceration (*arrow*) and intraplaque neovessels (*arrowheads*). MDCTA (**d**) confirming the presence of a small ulceration (*arrowhead*) in an otherwise smooth plaque causing stenosis of the internal carotid artery. Axial contrast-enhanced T1-weighted MRI image (**e**) of the same patient performed 1 week later for follow-up of stroke confirmed the presence of ulceration (*arrowhead*)

It is well known that ulcerations are not found in all carotid plaques with the same frequency but rather tend to occur in particular plaque types and are associated with specific histological findings. Namely, it was found that plaques with either ulcerations or simply irregular surfaces, based on DSA, were associated with rupture of the plaque’s fibrous cap, intraplaque haemorrhage, a large lipid core and less fibrous tissue [49]. These initial DSA findings warranted further research of ulcerated plaque’s composition with modern cross-sectional modalities [49]. Based on publications related to US and MDCTA, ulcerations were found to more often affect fatty plaques, less often fibrous and rarely calcified plaques [7]. From a pathogenic point of view, it is expected that ulceration is associated with intraplaque haemorrhage [50]. More precisely, MDCTA-detected ulceration is considered a risk factor for intraplaque haemorrhage [51]. In keeping with these findings, ulceration diagnosed on MDCTA is considered highly sensitive and specific for the presence of intraplaque haemorrhage as identified on MRI [52]. It was also observed that ulcerated plaques tend to be larger in volume and richer in lipid content [24, 53]. On the contrary, ulceration was found to be inversely associated with calcification [24]. Other studies could not confirm the association between ulceration and plaque volume, highlighting the fact that even smaller plaques may be ulcerated [53]. The association between ulceration and fatty hypoechoic plaques has also been reported with US [54]. Finally, it was observed that the plaque’s enhancement on MDCTA is strongly related with the presence of ulceration and neovascularisation [55, 56]. Indeed, studies with CEUS have demonstrated neovessels in close proximity to ulcerations [57] and that ulcerated plaques tend to have significantly more intraplaque neovessels compared to smooth plaques [58] (Figs. 6 and 7). In MRI studies, it was similarly concluded that lipid-necrotic content was a strong predictor for a new surface disruption in the form of either ulceration or fibrous cap rupture [59]. MRI studies confirmed the weak association between ulcerations and calcifications [59].

## Do ulcerated carotid plaques heal?

Once an ulcerated carotid plaque is diagnosed, a reasonable question occurring is how long will it take for it to heal. In general, it remains unknown how long an ulceration takes to heal although there are reports of cases healed after a few months [60]. MRI has been used to monitor the ulceration healing process in a dynamic way, demonstrating the creation of a new fibrous cap covering the ulcer cavity [60]. Other researchers have used 3D US to monitor ulcerated plaques prospectively for more than a year, reporting that the vast majority of plaques (76.5%) remained unchanged, 23.5% of ulcerations regressed while only 5.8% of ulcerations progressed [61]. In accordance with these results, another research group followed up carotid plaques with MDCTA for a period of approximately 2 years, concluding that 88% of plaques remained stable, 8% of plaques showed more surface irregularities while 4% of plaques were smoother. As for the ulcerated plaques, 67% of them showed no change on follow-up, while some of them regressed and only one progressed. This study also identified new ulcerations in previously non-ulcerated plaques [21].

## Imaging of ulceration

### Ultrasound

The discussion regarding the optimal imaging modality for diagnosis of carotid ulcerations has been ongoing since 1986 when US was considered superior to angiography for diagnosing ulceration [62]. Given the fact that US is the first-line modality for evaluation of carotid arteries, it is expected that this technique has been extensively investigated with conflicting results regarding the diagnostic accuracy for ulceration [32, 63–67]. Some studies showed high sensitivity and specificity [32, 65], while different researchers concluded that US is inadequate for diagnosing ulcers because of its low sensitivity (23–47%) [66, 68, 69], which was higher in plaques with <50% stenosis [69]. Similarly, correlation of US with histology was poor [70]. Beyond its low accuracy, US has been found to be characterised by low inter-observer agreement for ulceration [71].

Based on the technological advances in the field of US including modern transducers and image optimisation techniques, it is expected that current US devices may provide improved accuracy for diagnosing carotid ulceration [72]. The diversity in results regarding US accuracy may also be explained by the use of different diagnostic criteria and definitions for ulceration between different studies. Some studies have used flow reversal on colour Doppler for diagnosing ulceration [32]. The previously described de Bray criteria are among the most widely used in the literature [61, 71, 73–76]. Nevertheless, a recent study using histology as the reference method has shown that these criteria are only 35.7% sensitive and 75% specific [10]. The size criterion proved inaccurate, with histology detecting ulcers smaller than 2 mm and cavities larger than 2 mm not characterised as ulcers on histology. The newer criteria proposed were 85.7% sensitive and 81.3% specific, outperforming de Bray’s criteria [10]. Although US improved diagnostic accuracy with these new criteria, the acoustic shadow caused by calcified plaques still represents an inherent limitation of US [10]. The low echogenicity of the ulcer base compared to the nearby endothelium reflects the lower acoustic impedance of soft tissues such as thrombus compared to the plaque fibrous cap or normal endothelium. If this echogenicity criterion is not fulfilled, then a concavity on a plaque surface may not truly represent an ulcer but rather a simple cavity or even normal endothelium lying between two juxtapositioned plaques [10]. Other pitfalls in US interpretation potentially leading to false-positive results include mirror image artefact, which refers to the artefactual visualisation of flow within a plaque [76]. Beyond imaging the ulcer itself, modern US studies have evaluated new indirect findings such as a fine trembling motion of echogenic structures inside the plaque, which was found 93% sensitive and 60% specific [77].

### Contrast-enhanced ultrasound

CEUS has also been introduced in carotid arteries and investigated in plaque surface evaluation and identification of irregularities and ulcerations, showing better results than conventional US [14, 78–84]. A recent study compared US and CEUS in the diagnosis of carotid ulcerations having MDCTA as the reference method. CEUS outperformed US in terms of sensitivity, intra- and inter-reader agreement [13]. Similar subsequent studies have used CEUS for the detection of ulcerations in asymptomatic patients with diabetes [85]. Another recent study confirmed CEUS’s superiority to conventional US for diagnosing histological carotid plaque rupture. Using receiver-operating characteristic (ROC) analysis, the calculated optimal cut-off values of a cavity’s orifice, depth and width for the diagnosis of fibrous cap disruption were 1.4 mm, 1.3 mm and 1.88 mm respectively [86]. The B-flow technique is yet another US technique that can be used as an alternative to the Doppler technique and has been used for detection of carotid ulcers, with greater diagnostic accuracy than the colour Doppler technique [74, 87]. Using this technique, a swirling pattern of blood flow was demonstrated within ulcer cavities, a finding in keeping with previous experimental and US observations [33, 88]. Attempts to overcome ultrasound’s limitation of two-dimensional images are made with three-dimensional US, which as expected was found to detect more ulcers than conventional US [89, 90].

### Multi-detector computed tomography angiography

MDCTA constitutes a valuable modality for the evaluation of carotid disease, with accurate grading of stenosis and fewer complications compared to DSA [7, 12]. Early studies have shown good agreement with DSA for the detection of ulcerated plaques [91]. In keeping with these results, MDCTA showed good agreement with histology after endarterectomy [92]. Promising results were also found for MDCTA’s ability to characterise plaque’s composition compared with histology, although with less accuracy for the detection of ulcers according to some authors [93]. In a study comparing MDCTA, MRA and DSA, it was concluded that both MDCTA and MRA may replace DSA for accurate grading of stenosis. However, MDCTA detected luminal surface irregularities more often thanks to its excellent spatial resolution and, along with MRA, they proved superior to DSA for identification of ulcerations [94].

**Fig. 8:**
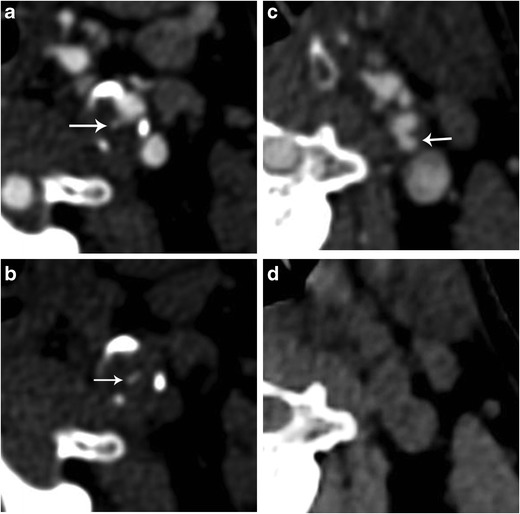
Differential diagnosis of ulceration and focal calcification with MDCTA. Axial MDCTA image (**a**) showing a potential small superficial ulceration (*arrow*). Respective unenhanced MDCT image (**b**) proving that this hyperdense material represents a focal calcification and not an ulcer. Axial MDCTA image (**c**) showing an ulcer (*arrow*). The unenhanced MDCT image (**d**) confirms the absence of calcification at this point of the vessel

Studies comparing US and MDCTA have shown little agreement between these methods for both the diagnosis of ulcerations and characterisation of plaques as smooth or irregular [95]. With histology as the reference method, MDCTA proved superior to US, with 93% sensitivity and 98% specificity for diagnosing ulceration whereas the latter was only 37% sensitive and 91% specific [7, 96]. Improved accuracy found in this study reflected the use of multidetector technology in comparison with other studies assessing single-detector CT [93, 97, 98]. MDCTA’s ability to readily detect ulcers is partly attributed to the availability of specialised three-dimensional reformatting software such as multiplanar reconstruction (MPR), maximum intensity projection (MIP) and volume rendering (VR). MDCTA is not free of limitations though, as thorough evaluation may be hindered by artefacts including beam hardening in heavily calcified plaques [7], which may hide small ulcerations [7, 99, 100]. In a study comparing various MDCTA techniques for the detection of ulceration, axial images and VR proved to be the most accurate. The overall accuracy of MDCTA, with all techniques deployed, showed 93.9% sensitivity and 98.7% specificity [101]. When reviewing carotid MDCTA scans, it should be kept in mind that hyperdense material projecting outside the vascular lumen may represent either a focal calcification or a true ulceration. To differentiate these entities, a density threshold of 600 Hounsfield units (HU) has been used [13]. Ideally, though, an unenhanced scan should be performed prior to the intravenous contrast administration (Fig. 8).

Conventional angiography is traditionally considered the reference method for grading carotid stenosis although its accuracy for the detection of ulcerations has been questioned [94, 98, 102–104]. Moreover, it represents an interventional technique associated with a periprocedural risk for thromboembolic events [105]. As a result, modern non-invasive modalities such as MDCTA [97, 98] or MRA [106, 107] are gradually replacing DSA [7]. Anzidei et al. compared US, MDCTA and MRA with DSA, concluding that MDCTA has excellent diagnostic accuracy with a sensitivity, specificity, positive and negative predictive value up to 100% for the diagnosis of ulceration, outperforming MRA [108].

### Magnetic resonance angiography

**Fig. 9:**
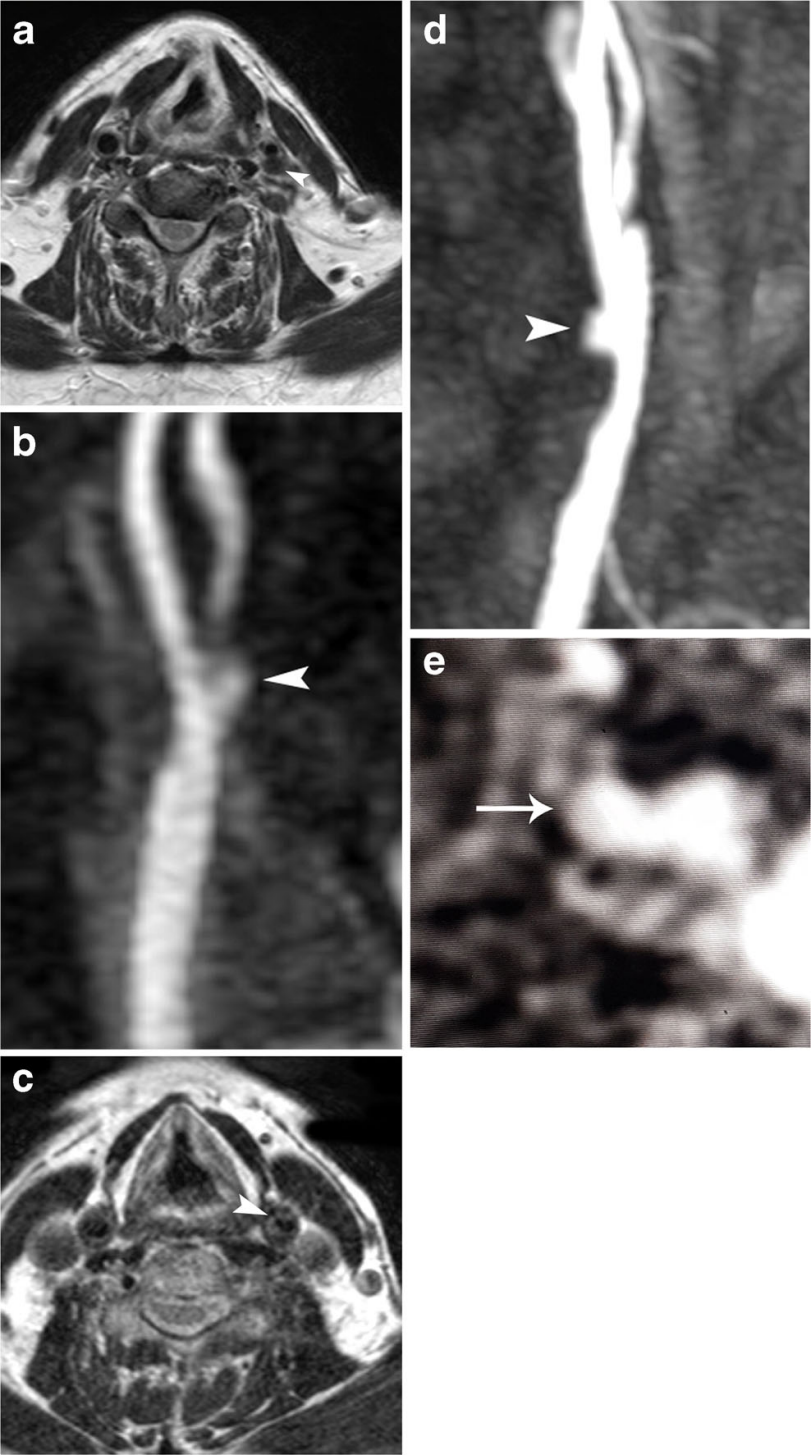
MRA findings in ulcerated carotid plaques. Axial T2-weighted image (**a**) showing flow void in the vessel lumen and slightly higher signal intensity within the origin of internal carotid artery, possibly representing a stenotic plaque (*arrowhead*). Contrast-enhanced MRA (**b**) identifying a type 3 ulcerated carotid plaque (arrowhead, same patient with Fig. 4**d**). Axial T2-weighted image (**c**) showing a projection of the luminal flow void within a high-signal intensity area, consistent with an ulcerated fatty plaque (*arrowhead*). Contrast-enhanced MRA (**d**) confirming the presence of a type 1a ulceration (arrowhead, same patient as in Fig. 4**a**). Axial contrast-enhanced T1-weighted image confirming the presence of contrast material within the ulcer’s cavity (*arrow*) (**e**)

Magnetic resonance imaging (MRI) has also been used for the diagnosis of ulcerated carotid plaques with good inter-observer agreement [109]. Longitudinal black blood cardiovascular MRA improves the technique’s sensitivity (80%) and specificity (82.3%) for the detection of ulceration in comparison with simple evaluation of axial images [110]. Contrast-enhanced MRA (CEMRA) is considered superior to time-of-flight (TOF) MRA for ulceration, which had more false-negative results (Fig. 9). The main reasons explaining TOF-MRA’s lower accuracy include the ulcer’s orientation and location in relation to the point of maximum stenosis and geometry in the form of neck-to-depth ratio [111]. MRI’s particular strength in detection of ulcerations relies on its ability to image the plaque’s fibrous cap as a black zone lying between the bright lumen and the grey plaque content. Absence of this dark zone represents rupture of the fibrous cap and thus ulceration [111, 112]. If blood pool agents were used, then CE-MRA was found superior to MDCTA both for grading of stenosis and characterisation of plaque morphology [113].

## Conclusion

Carotid plaque surface morphology represents an important feature of plaque vulnerability as both surface irregularity and ulceration have been correlated with stroke. The diagnosis of carotid ulceration relies on imaging and virtually all modalities have been investigated in that respect, showing varying degrees of diagnostic accuracy. US as the first-line imaging modality has the potential to detect ulcerations, especially if microbubbles are used. Nevertheless, cross-sectional modalities such as MDCTA and CEMRA have proved valuable for the delineation of carotid plaque surface and diagnosis of ulceration.

## References

[1] Fairhead JF, Rothwell PM (2005). The need for urgency in identification and treatment of symptomatic carotid stenosis is already established. Cerebrovasc Dis.

[2] Bonati LH, Nederkoorn PJ (2016). Clinical perspective of carotid plaque imaging. Neuroimaging Clin N Am.

[3] Qureshi AI, Alexandrov AV, Tegeler CH (2007). Guidelines for screening of extracranial carotid artery disease: a statement for healthcare professionals from the multidisciplinary practice guidelines committee of the American Society of Neuroimaging; cosponsored by the Society of Vascular and Interventional Neurology. J Neuroimaging.

[4] Saba L, Anzidei M, Marincola BC (2014). Imaging of the carotid artery vulnerable plaque. Cardiovasc Intervent Radiol.

[5] Adams HP, Bendixen BH, Kappelle LJ (1993). Classification of subtype of acute ischemic stroke. Definitions for use in a multicenter clinical trial. TOAST. Trial of Org 10172 in acute stroke treatment. Stroke.

[6] Saba L, Anzidei M, Sanfilippo R (2012). Imaging of the carotid artery. Atherosclerosis.

[7] Saba L, Caddeo G, Sanfilippo R, Montisci R, Mallarini G (2007). CT and ultrasound in the study of ulcerated carotid plaque compared with surgical results: potentialities and advantages of multidetector row CT angiography. AJNR Am J Neuroradiol.

[8] Eckstein HH, Kuhnl A, Dorfler A (2013). The diagnosis, treatment and follow-up of extracranial carotid stenosis. Dtsch Arztebl Int.

[9] Grant EG, Benson CB, Moneta GL (2003). Carotid artery stenosis: gray-scale and Doppler US diagnosis—Society of Radiologists in Ultrasound Consensus Conference. Radiology.

[10] Muraki M, Mikami T, Yoshimoto T (2012). New criteria for the sonographic diagnosis of a plaque ulcer in the extracranial carotid artery. AJR Am J Roentgenol.

[11] Leffers AM, Wagner A (2000). Neurologic complications of cerebral angiography. A retrospective study of complication rate and patient risk factors. Acta Radiol.

[12] Berg M, Zhang Z, Ikonen A (2005). Multi-detector row CT angiography in the assessment of carotid artery disease in symptomatic patients: comparison with rotational angiography and digital subtraction angiography. AJNR Am J Neuroradiol.

[13] ten Kate GL, van Dijk AC, van den Oord SC (2013). Usefulness of contrast-enhanced ultrasound for detection of carotid plaque ulceration in patients with symptomatic carotid atherosclerosis. Am J Cardiol.

[14] Saha SA, Gourineni V, Feinstein SB (2016). The use of contrast-enhanced ultrasonography for imaging of carotid atherosclerotic plaques: current evidence, future directions. Neuroimaging Clin N Am.

[15] de Bray JM, Baud JM, Dauzat M (1997). Consensus concerning the morphology and the risk of carotid plaques. Cerebrovasc Dis.

[16] Sitzer M, Muller W, Siebler M (1995). Plaque ulceration and lumen thrombus are the main sources of cerebral microemboli in high-grade internal carotid artery stenosis. Stroke.

[17] Brinjikji W, Rabinstein AA, Lanzino G (2015). Ultrasound characteristics of symptomatic carotid plaques: a systematic review and meta-analysis. Cerebrovasc Dis.

[18] Eyding J, Geier B, Staub D (2011). Current strategies and possible perspectives of ultrasonic risk stratification of ischemic stroke in internal carotid artery disease. Ultraschall Med.

[19] Kuk M, Wannarong T, Beletsky V, Parraga G, Fenster A, Spence JD (2014). Volume of carotid artery ulceration as a predictor of cardiovascular events. Stroke.

[20] de Weert TT, Cretier S, Groen HC (2009). Atherosclerotic plaque surface morphology in the carotid bifurcation assessed with multidetector computed tomography angiography. Stroke.

[21] van Gils MJ, Homburg PJ, Rozie S, de Weert TT, Dippel DW, van der Lugt A (2011). Evolution of atherosclerotic carotid plaque morphology: do ulcerated plaques heal? A serial multidetector CT angiography study. Cerebrovasc Dis.

[22] Kim DI, Lee SJ, Lee BB (2000). The relationship between the angiographic findings and the clinical features of carotid artery plaque. Surg Today.

[23] Lovett JK, Rothwell PM (2003). Site of carotid plaque ulceration in relation to direction of blood flow: an angiographic and pathological study. Cerebrovasc Dis.

[24] Homburg PJ, Rozie S, van Gils MJ (2011). Association between carotid artery plaque ulceration and plaque composition evaluated with multidetector CT angiography. Stroke.

[25] Fisher M, Paganini-Hill A, Martin A (2005). Carotid plaque pathology: thrombosis, ulceration, and stroke pathogenesis. Stroke.

[26] Golledge J, Greenhalgh RM, Davies AH (2000). The symptomatic carotid plaque. Stroke.

[27] Wechsler LR (1988). Ulceration and carotid artery disease. Stroke.

[28] Moore WS, Hall AD (1968). Ulcerated atheroma of the carotid artery. Am J Surg.

[29] Kessler C, von Maravic M, Bruckmann H, Kompf D (1995). Ultrasound for the assessment of the embolic risk of carotid plaques. Acta Neurol Scand.

[30] Jovanovic ZB, Pavlovic MA, Vujisic Tesic PB (2013). The significance of the ultrasound diagnostics in evaluation of the emboligenic pathogenesis of transient ischemic attacks. Ultrasound Med Biol.

[31] Imbesi SG, Kerber CW (1999). An experimental and angiographic explanation of why ulcerated carotid bulbs embolize. Interv Neuroradiol.

[32] Fürst H, Hartl WH, Jansen I, Liepsch D, Lauterjung L, Schildberg FW (1992). Color-flow Doppler sonography in the identification of ulcerative plaques in patients with high-grade carotid artery stenosis. AJNR Am J Neuroradiol.

[33] Rafailidis V, Charitanti A, Tegos T, Rafailidis D, Chryssogonidis I (2016). Swirling of microbubbles: demonstration of a new finding of carotid plaque ulceration on contrast-enhanced ultrasound explaining the arterio-arterial embolism mechanism. Clin Hemorheol Microcirc.

[34] Valton L, Larrue V, Arrue P, Geraud G, Bes A (1995). Asymptomatic cerebral embolic signals in patients with carotid stenosis. Correlation with appearance of plaque ulceration on angiography. Stroke.

[35] Orlandi G, Parenti G, Landucci Pellegrini L (1999). Plaque surface and microembolic signals in moderate carotid stenosis. Ital J Neurol Sci.

[36] Rothwell PM, Gibson R, Warlow CP (2000). Interrelation between plaque surface morphology and degree of stenosis on carotid angiograms and the risk of ischemic stroke in patients with symptomatic carotid stenosis. On behalf of the European Carotid Surgery Trialists’ Collaborative Group. Stroke.

[37] Eliasziw M, Streifler JY, Fox AJ, Hachinski VC, Ferguson GG, Barnett HJ (1994). Significance of plaque ulceration in symptomatic patients with high-grade carotid stenosis. North American Symptomatic Carotid Endarterectomy Trial. Stroke.

[38] Ballotta E, Angelini A, Mazzalai F, Piatto G, Toniato A, Baracchini C (2014). Carotid endarterectomy for symptomatic low-grade carotid stenosis. J Vasc Surg.

[39] Tegos TJ, Kalodiki E, Daskalopoulou SS, Nicolaides AN (2000). Stroke: epidemiology, clinical picture, and risk factors—Part I of III. Angiology.

[40] Brajovic MD, Markovic N, Loncar G (2009). The influence of various morphologic and hemodynamic carotid plaque characteristics on neurological events onset and deaths. Sci World J.

[41] Singh TD, Kramer CL, Mandrekar J, Lanzino G, Rabinstein AA (2015). Asymptomatic carotid stenosis: risk of progression and development of symptoms. Cerebrovasc Dis.

[42] Gao P, Chen ZQ, Jiao LQ, Ling F (2007). The correlation of carotid plaque pathohistologic features and neurological symptoms: a meta-analysis of observational studies. Neurol India.

[43] Handa N, Matsumoto M, Maeda H, Hougaku H, Kamada T (1995). Ischemic stroke events and carotid atherosclerosis. Results of the Osaka Follow-up Study for Ultrasonographic Assessment of Carotid Atherosclerosis (the OSACA Study). Stroke.

[44] Nakamura T, Tsutsumi Y, Shimizu Y, Uchiyama S (2013). Ulcerated carotid plaques with ultrasonic echolucency are causatively associated with thromboembolic cerebrovascular events. J Stroke Cerebrovasc Dis.

[45] North American Symptomatic Carotid Endarterectomy Trial Collaborators (1991). Beneficial effect of carotid endarterectomy in symptomatic patients with high-grade carotid stenosis. N Engl J Med.

[46] Hokari M, Kuroda S, Yasuda H (2011). Lumen morphology in mild-to-moderate internal carotid artery stenosis correlates with neurological symptoms. J Neuroimaging.

[47] Prabhakaran S, Rundek T, Ramas R (2006). Carotid plaque surface irregularity predicts ischemic stroke: the northern Manhattan study. Stroke.

[48] Naylor AR, Sillesen H, Schroeder TV (2015). Clinical and imaging features associated with an increased risk of early and late stroke in patients with symptomatic carotid disease. Eur J Vasc Endovasc Surg.

[49] Lovett JK, Gallagher PJ, Hands LJ, Walton J, Rothwell PM (2004). Histological correlates of carotid plaque surface morphology on lumen contrast imaging. Circulation.

[50] van Dijk AC, Truijman MT, Hussain B (2015). Intraplaque hemorrhage and the plaque surface in carotid atherosclerosis: The Plaque At RISK Study (PARISK). AJNR Am J Neuroradiol.

[51] McLaughlin MS, Hinckley PJ, Treiman SM (2015). Optimal prediction of carotid intraplaque hemorrhage using clinical and lumen imaging markers. AJNR Am J Neuroradiol.

[52] U-King-Im JM, Fox AJ, Aviv RI (2010). Characterization of carotid plaque hemorrhage: a CT angiography and MR intraplaque hemorrhage study. Stroke.

[53] Saba L, Sanfilippo R, Sannia S (2012). Association between carotid artery plaque volume, composition, and ulceration: a retrospective assessment with MDCT. AJR Am J Roentgenol.

[54] Gray-Weale AC, Graham JC, Burnett JR, Byrne K, Lusby RJ (1988). Carotid artery atheroma: comparison of preoperative B-mode ultrasound appearance with carotid endarterectomy specimen pathology. J Cardiovasc Surg.

[55] Saba L, Lai ML, Montisci R (2012). Association between carotid plaque enhancement shown by multidetector CT angiography and histologically validated microvessel density. Eur Radiol.

[56] Saba L, Tamponi E, Raz E (2014). Correlation between fissured fibrous cap and contrast enhancement: preliminary results with the use of CTA and histologic validation. AJNR Am J Neuroradiol.

[57] Vicenzini E, Giannoni MF, Puccinelli F (2007). Detection of carotid adventitial vasa vasorum and plaque vascularization with ultrasound cadence contrast pulse sequencing technique and echo-contrast agent. Stroke.

[58] van den Oord SC, Akkus Z, Roeters van Lennep JE (2013). Assessment of subclinical atherosclerosis and intraplaque neovascularization using quantitative contrast-enhanced ultrasound in patients with familial hypercholesterolemia. Atherosclerosis.

[59] Underhill HR, Yuan C, Yarnykh VL (2010). Predictors of surface disruption with MR imaging in asymptomatic carotid artery stenosis. AJNR Am J Neuroradiol.

[60] Qiao Y, Farber A, Semaan E, Hamilton JA (2008). Images in cardiovascular medicine. Healing of an asymptomatic carotid plaque ulceration. Circulation.

[61] Schminke U, Motsch L, Hilker L, Kessler C (2000). Three-dimensional ultrasound observation of carotid artery plaque ulceration. Stroke.

[62] Bornmyr S, Jugquist G, Olivecrona H, Takolander R, Bergqvist D, Lindell SE (1986). Ulceration of the carotid bifurcation. A preliminary report on a diagnostic problem. Acta Chir Scand.

[63] Johnson JM, Ansel AL, Morgan S, DeCesare D (1982). Ultrasonographic screening for evaluation and follow-up of carotid artery ulceration. A new basis for assessing risk. Am J Surg.

[64] Connolly JE, Brownell DA, Levine EF, McCart PM (1985). Accuracy and indications of diagnostic studies for extracranial carotid disease. Arch Surg.

[65] O’Donnell TF, Erdoes L, Mackey WC (1985). Correlation of B-mode ultrasound imaging and arteriography with pathologic findings at carotid endarterectomy. Arch Surg.

[66] Friedrich JM, Arlart IP, Schumacher KA, Hamann H (1987). Role of digitalized angiography by venous route in the study of carotid bifurcation. Value in the diagnosis of ulcerated lesions. J Radiol.

[67] Hansen F, Bergqvist D, Eriksson A, Maly P, Takolander R (1989). Evaluation of ulceration in the extracranial carotid artery with ultrasonography: a comparison with arteriography. Eur J Vasc Surg.

[68] Anderson DC, Loewenson R, Yock D, Farber R, Larson D, Bromer M (1983). B-mode, real-time carotid ultrasonic imaging. Correlation with angiography. Arch Neurol.

[69] Comerota AJ, Katz ML, White JV, Grosh JD (1990). The preoperative diagnosis of the ulcerated carotid atheroma. J Vasc Surg.

[70] Snow M, Ben-Sassi A, Winter RK (2007). Can carotid ultrasound predict plaque histopathology?. J Cardiovasc Surg.

[71] Sitzer M, Muller W, Rademacher J (1996). Color-flow Doppler-assisted duplex imaging fails to detect ulceration in high-grade internal carotid artery stenosis. J Vasc Surg.

[72] Hu CH, Xu XC, Cannata JM, Yen JT, Shung KK (2006). Development of a real-time, high-frequency ultrasound digital beamformer for high-frequency linear array transducers. IEEE Trans Ultrason Ferroelectr Freq Control.

[73] Polak JF (2001). Carotid ultrasound. Radiol Clin N Am.

[74] Reiter M, Horvat R, Puchner S (2007). Plaque imaging of the internal carotid artery - correlation of B-flow imaging with histopathology. AJNR Am J Neuroradiol.

[75] Hartmann A, Mohr JP, Thompson JL, Ramos O, Mast H (1999). Interrater reliability of plaque morphology classification in patients with severe carotid artery stenosis. Acta Neurol Scand.

[76] Arning C, Eckert B (2004). The diagnostic relevance of colour Doppler artefacts in carotid artery examinations. Eur J Radiol.

[77] Muraki M, Mikami T, Yoshimoto T (2016). Sonographic detection of abnormal plaque motion of the carotid artery: its usefulness in diagnosing high-risk lesions ranging from plaque rupture to ulcer formation. Ultrasound Med Biol.

[78] Partovi S, Loebe M, Aschwanden M (2012). Contrast-enhanced ultrasound for assessing carotid atherosclerotic plaque lesions. AJR Am J Roentgenol.

[79] Schinkel AF, Kaspar M, Staub D (2016). Contrast-enhanced ultrasound: clinical applications in patients with atherosclerosis. Int J Cardiovasc Imaging.

[80] Rafailidis V, Pitoulias G, Kouskouras K, Rafailidis D (2015). Contrast-enhanced ultrasonography of the carotids. Ultrasonography.

[81] Kono Y, Pinnell SP, Sirlin CB (2004). Carotid arteries: contrast-enhanced US angiography—preliminary clinical experience. Radiology.

[82] Piscaglia F, Nolsoe C, Dietrich CF (2012). The EFSUMB guidelines and recommendations on the clinical practice of contrast enhanced ultrasound (CEUS): update 2011 on non-hepatic applications. Ultraschall Med.

[83] Sirlin CB, Lee YZ, Girard MS (2001). Contrast-enhanced B-mode US angiography in the assessment of experimental in vivo and in vitro atherosclerotic disease. Acad Radiol.

[84] Rafailidis V, Charitanti A, Tegos T, Destanis E, Chryssogonidis I (2016) Contrast-enhanced ultrasound of the carotid system: a review of the current literature. J Ultrasound. doi:10.1007/s40477-017-0239-4 10.1007/s40477-017-0239-4 PMC5440332 28592999

[85] van den Oord SC, Akkus Z, Renaud G (2014). Assessment of carotid atherosclerosis, intraplaque neovascularization, and plaque ulceration using quantitative contrast-enhanced ultrasound in asymptomatic patients with diabetes mellitus. Eur Heart J Cardiovasc Imaging.

[86] Hamada O, Sakata N, Ogata T, Shimada H, Inoue T (2016). Contrast-enhanced ultrasonography for detecting histological carotid plaque rupture: quantitative analysis of ulcer. Int J Stroke.

[87] Jung EM, Kubale R, Ritter G (2007). Diagnostics and characterisation of preocclusive stenoses and occlusions of the internal carotid artery with B-flow. Eur Radiol.

[88] Umemura A, Yamada K (2001). B-mode flow imaging of the carotid artery. Stroke.

[89] Igase K, Kumon Y, Matsubara I (2015). Utility of 3-dimensional ultrasound imaging to evaluate carotid artery stenosis: comparison with magnetic resonance angiography. J Stroke Cerebrovasc Dis.

[90] Heliopoulos J, Vadikolias K, Piperidou C, Mitsias P (2011). Detection of carotid artery plaque ulceration using 3-dimensional ultrasound. J Neuroimaging.

[91] Anderson GB, Ashforth R, Steinke DE, Ferdinandy R, Findlay JM (2000). CT angiography for the detection and characterization of carotid artery bifurcation disease. Stroke.

[92] Wintermark M, Jawadi SS, Rapp JH (2008). High-resolution CT imaging of carotid artery atherosclerotic plaques. AJNR Am J Neuroradiol.

[93] Oliver TB, Lammie GA, Wright AR (1999). Atherosclerotic plaque at the carotid bifurcation: CT angiographic appearance with histopathologic correlation. AJNR Am J Neuroradiol.

[94] Randoux B, Marro B, Koskas F (2001). Carotid artery stenosis: prospective comparison of CT, three-dimensional gadolinium-enhanced MR, and conventional angiography. Radiology.

[95] Saba L, Sanfilippo R, Montisci R, Atzeni M, Ribuffo D, Mallarini G (2011). Vulnerable plaque: detection of agreement between multi-detector-row CT angiography and US-ECD. Eur J Radiol.

[96] Vucaj-Cirilovic V, Lucic M, Petrovic K, Nikolic O, Govorcin M, Stojanovic S (2011). Color Doppler ultrasonography and multislice computer tomography angiography in carotid plaque detection and characterization. Vojnosanit Pregl.

[97] Debernardi S, Martincich L, Lazzaro D, Comelli S, Raso AM, Regge D (2004). CT angiography in the assessment of carotid atherosclerotic disease: results of more than two years’ experience. Radiol Med.

[98] Walker LJ, Ismail A, McMeekin W, Lambert D, Mendelow AD, Birchall D (2002). Computed tomography angiography for the evaluation of carotid atherosclerotic plaque: correlation with histopathology of endarterectomy specimens. Stroke.

[99] Schwartz RB, Jones KM, Chernoff DM (1992). Common carotid artery bifurcation: evaluation with spiral CT. Work in progress. Radiology.

[100] Cumming MJ, Morrow IM (1994). Carotid artery stenosis: a prospective comparison of CT angiography and conventional angiography. AJR Am J Roentgenol.

[101] Saba L, Caddeo G, Sanfilippo R, Montisci R, Mallarini G (2007). Efficacy and sensitivity of axial scans and different reconstruction methods in the study of the ulcerated carotid plaque using multidetector-row CT angiography: comparison with surgical results. AJNR Am J Neuroradiol.

[102] Runge VM, Kirsch JE, Lee C (1993). Contrast-enhanced MR angiography. J Magn Reson Imaging.

[103] Ricotta JJ, Aburahma A, Ascher E (2011). Updated society for vascular surgery guidelines for management of extracranial carotid disease. J Vasc Surg.

[104] Streifler JY, Eliasziw M, Fox AJ (1994). Angiographic detection of carotid plaque ulceration. Comparison with surgical observations in a multicenter study. North American Symptomatic Carotid Endarterectomy Trial. Stroke.

[105] Hessel SJ, Adams DF, Abrams HL (1981). Complications of angiography. Radiology.

[106] Modaresi KB, Cox TC, Summers PE (1999). Comparison of intra-arterial digital subtraction angiography, magnetic resonance angiography and duplex ultrasonography for measuring carotid artery stenosis. Br J Surg.

[107] El-Saden SM, Grant EG, Hathout GM, Zimmerman PT, Cohen SN, Baker JD (2001). Imaging of the internal carotid artery: the dilemma of total versus near total occlusion. Radiology.

[108] Anzidei M, Napoli A, Zaccagna F (2012). Diagnostic accuracy of colour Doppler ultrasonography, CT angiography and blood-pool-enhanced MR angiography in assessing carotid stenosis: a comparative study with DSA in 170 patients. Radiol Med.

[109] den Hartog AG, Bovens SM, Koning W (2013). Current status of clinical magnetic resonance imaging for plaque characterisation in patients with carotid artery stenosis. Eur J Vasc Endovasc Surg.

[110] Yu W, Underhill HR, Ferguson MS (2009). The added value of longitudinal black-blood cardiovascular magnetic resonance angiography in the cross sectional identification of carotid atherosclerotic ulceration. J Cardiovasc Magn Reson.

[111] Etesami M, Hoi Y, Steinman DA (2013). Comparison of carotid plaque ulcer detection using contrast-enhanced and time-of-flight MRA techniques. AJNR Am J Neuroradiol.

[112] Brinjikji W, Huston J, Rabinstein AA, Kim GM, Lerman A, Lanzino G (2016). Contemporary carotid imaging: from degree of stenosis to plaque vulnerability. J Neurosurg.

[113] Anzidei M, Napoli A, Geiger D (2010). Preliminary experience with MRA in evaluating the degree of carotid stenosis and plaque morphology using high-resolution sequences after gadofosveset trisodium (Vasovist) administration: comparison with CTA and DSA. Radiol Med.

